# Immune Response Against SARS-CoV-2

**DOI:** 10.3390/vaccines12121390

**Published:** 2024-12-11

**Authors:** Angela Stufano, Valentina Schino, Guglielmo Lucchese

**Affiliations:** 1 Interdisciplinary Department of Medicine, University of Bari Aldo Moro, 70124 Bari, Italy; angela.stufano@uniba.it; 2 Department of Experimental Medicine, University of Salento, Via Lecce-Monteroni, 73047 Lecce, Italy; guglielmo.lucchese@unisalento.it; 3 Department of Neurology, University Medicine Greifswald, Ferdinand-Sauerbruch-Straße, 17475 Greifswald, Germany

The immune response to SARS-CoV-2 infection is highly complex, involving a dynamic interplay between the innate and adaptive immune systems. Understanding this interplay is crucial for deciphering the varying disease outcomes, from mild to severe COVID-19. This editorial provides a concise overview of recent developments in the field, identifies the key gaps in knowledge addressed by the Special Issue, and outlines future research directions.

SARS-CoV-2 initially triggers an innate immune response through pattern recognition receptors (PRRs), which detect viral proteins and RNA, leading to the release of key pro-inflammatory cytokines such as interleukin-6 (IL-6), tumor necrosis factor alpha (TNFα), and type I and III interferons (IFNs) [1,2]. However, the virus employs various strategies to evade this response, including inhibiting interferon signaling, impairing IFN release, and suppressing cytokine production. These mechanisms can contribute to severe disease outcomes, such as cytokine storms [3].

The adaptive immune response is critical, with antigen-presenting cells (APCs) presenting viral antigens to T and B cells. This interaction results in the production of virus-specific antibodies and the activation of cytotoxic T cells. CD4+ T helper cells facilitate B cell differentiation into antibody-producing plasma cells, while CD8+ cytotoxic T cells directly target and eliminate infected cells. Yet, SARS-CoV-2 has evolved mechanisms—such as reduced MHC-I expression and immune cell exhaustion—that impair long-lasting immunity and immune memory [4,5]. Variants of the virus, like Omicron, have shown reduced sensitivity to certain immune responses, which affects the efficacy of immunity from vaccines and monoclonal antibodies [6].

The link between SARS-CoV-2 infection and autoimmunity is particularly concerning. Post-infection, individuals are at an increased risk of developing systemic autoimmune diseases and long-term neurological symptoms. This is thought to result from immune dysregulation, including the production of autoantibodies targeting various tissues, such as the brain and nervous system [7,8]. These autoantibodies, possibly triggered by molecular mimicry, contribute to severe disease and lingering complications like memory deficits, dysautonomia, and chronic pain [8,9,10]. Early detection and targeted therapeutic strategies are crucial to managing these long-term consequences of COVID-19.

Managing severe symptoms and long COVID often involves therapeutic interventions to modulate the inflammatory response. Anti-inflammatory drugs like dexamethasone and IL-6 inhibitors (e.g., tocilizumab) help reduce cytokine storms [7]. Precision medicine and personalized treatments have proven beneficial for managing long COVID, a chronic condition characterized by persistent inflammation and immune dysregulation [11].

The findings from studies in this Special Issue underscore the multifaceted nature of the immune response to SARS-CoV-2. They highlight its implications for vaccine development, therapeutic strategies, and long-term management plans for both acute COVID-19 and long COVID. For instance, Li et al. investigated immunity persistence post-vaccination, demonstrating the importance of booster doses in regions with high incidence of Omicron. Zalewska et al. explored immune responses in individuals receiving a heterologous ChAdOx1/BNT162b2 booster, noting enhanced antibody titers and IFN-γ responses. Kibler et al. reported on a novel intranasal vaccine, which offered full protection against a lethal challenge with a heavily mutated strain in mice, highlighting the potential for mucosal immunity to combat SARS-CoV-2 variants. Vega-Magaña and colleagues examined T-cell responses in vaccinated individuals, revealing a decline in neutralizing antibodies over six months post-vaccination, which emphasizes the role of cellular immunity alongside humoral responses. Pourmasumi et al. reviewed the impact of SARS-CoV-2 on male fertility, discussing the alterations in sperm parameters post-infection, while Hajissa et al. reviewed SARS-CoV-2 antibodies’ role in diagnostics and vaccine development. Karimabad et al. explored chemokines in the immune response and pathogenesis of COVID-19, suggesting their potential as therapeutic targets and markers for vaccine-induced immunity.

Each study in this collection provides unique insights into various aspects of COVID-19 immunity, vaccine efficacy, and broader health impacts. These findings are crucial for understanding pandemic management and immunological preventive approaches for severe disease and long-term sequelae. The complexity of the immune response to SARS-CoV-2 highlights the need for a multifaceted public health approach that integrates prevention, acute care, and chronic disease management. Future research should focus on elucidating the precise mechanisms of immune evasion by SARS-CoV-2, optimizing vaccine strategies to enhance long-lasting immunity, and developing therapies to prevent and manage long COVID. These efforts will be critical for addressing the ongoing challenges posed by the COVID-19 pandemic.

In summary, the immune response to SARS-CoV-2 is highly complex, involving both innate and adaptive immunity, and shaped by the virus’s immune evasion strategies. The contributions to this Special Issue provide a deeper understanding of this evolving relationship, guiding vaccine development, therapeutic strategies, and long-term management plans for both acute COVID-19 and long COVID. These insights emphasize the importance of a holistic public health approach to managing the COVID-19 pandemic, with a focus on both prevention and treatment. Future research must continue to explore the dynamics of SARS-CoV-2 immunity and the implications for public health policy and clinical practice.
